# Variation in COVID-19 Mortality in the US by Race and Ethnicity and Educational Attainment

**DOI:** 10.1001/jamanetworkopen.2021.35967

**Published:** 2021-11-23

**Authors:** Justin M. Feldman, Mary T. Bassett

**Affiliations:** 1 FXB Center for Health and Human Rights, Harvard T.H. Chan School of Public Health, Boston, Massachusetts

## Abstract

**Importance:**

Racial and ethnic inequities in COVID-19 mortality have been well documented, but little prior research has assessed the combined roles of race and ethnicity and educational attainment.

**Objective:**

To measure inequality in COVID-19 mortality jointly by race and ethnicity and educational attainment.

**Design, Setting, and Participants:**

This cross-sectional study analyzed data on COVID-19 mortality from the 50 US states and the District of Columbia for the full calendar year 2020. It included all persons in the United States aged 25 years or older and analyzed them in subgroups jointly stratified by age, sex, race and ethnicity, and educational attainment.

**Main Outcomes and Measures:**

Population-based cumulative mortality rates attributed to COVID-19.F

**Results:**

Among 219.1 million adults aged 25 years or older (113.3 million women [51.7%]; mean [SD] age, 51.3 [16.8] years), 376 125 COVID-19 deaths were reported. Age-adjusted cumulative mortality rates per 100 000 ranged from 54.4 (95% CI, 49.8-59.0 per 100 000 population) among Asian women with some college to 699.0 (95% CI, 612.9-785.0 per 100 000 population) among Native Hawaiian and Other Pacific Islander men with a high school degree or less. Racial and ethnic inequalities in COVID-19 mortality rates remained when comparing within educational attainment categories (median rate ratio reduction, 17% [IQR, 0%-25%] for education-stratified estimates vs unstratified, with non-Hispanic White individuals as the reference). If all groups had experienced the same mortality rates as college-educated non-Hispanic White individuals, there would have been 48% fewer COVID-19 deaths among adults aged 25 years or older overall, including 71% fewer deaths among racial and ethnic minority populations and 89% fewer deaths among racial and ethnic minority populations aged 25 to 64 years.

**Conclusions and Relevance:**

Public health research and practice should attend to the ways in which populations that share socioeconomic characteristics may still experience racial and ethnic inequity in the distribution of risk factors for SARS-CoV-2 exposure and infection fatality rates (eg, housing, occupation, and prior health status). This study suggests that a majority of deaths among racial and ethnic minority populations could have been averted had all groups experienced the same mortality rate as college-educated non-Hispanic White individuals, thus highlighting the importance of eliminating joint racial-socioeconomic health inequities.

## Key Points

**Question:**

How did COVID-19 mortality rates during 2020 in the United States vary by race and ethnicity in combination with educational attainment?

**Findings:**

In this cross-sectional study of 219.1 million adults aged 25 years or older, most racial and ethnic minority populations had higher age-adjusted mortality rates than non-Hispanic White populations, including when comparing within levels of educational attainment. If all racial and ethnic populations had experienced the same mortality rates as college-educated non-Hispanic White populations, 71% fewer deaths among racial and ethnic minority populations would have occurred.

**Meaning:**

This study suggests that public health research and practice should attend to the ways in which populations that share socioeconomic characteristics may still experience racial and ethnic inequity in COVID-19 mortality rates.

## Teaser

This cross-sectional study measures inequality in US COVID-19 mortality jointly by race and ethnicity and educational attainment.

## Introduction

Numerous studies have documented racial and ethnic inequities in COVID-19 mortality rates in the United States.^1,2,3^ Racial and ethnic inequalities are large, with cumulative mortality rate ratios (MRRs) exceeding those of other major causes of death.^3^ However, to our knowledge, few studies have assessed COVID-19 mortality inequities by race and ethnicity in combination with socioeconomic position, and the data to conduct such analyses on the national level have been unavailable until recently. One US-wide study found roughly equal cumulative mortality rates by race and ethnicity within educational attainment categories, but the data source did not allow for age standardization and, as the authors noted, the results were likely to underestimate racial and ethnic inequities because US White populations have distributions that skew older.^2^ That study, along with another analysis of California’s Latinx population,^4^ found that persons in the lowest socioeconomic position experienced the highest COVID-19 mortality rates within racial and ethnic groups.

Our study uses a recently available public data set on COVID-19 mortality, which permits subgroup analysis by race and ethnicity, educational attainment, age, and sex, and therefore allows for a more complete examination of racialized socioeconomic inequities than previous studies. Our research is informed by prior literature that has illustrated how racialized differences in health are not reducible to inequalities in educational attainment, and involve multiple other pathways (eg, medical discrimination, inequitable treatment by the criminal justice system, and environmental injustice).^5^ We assessed 3 hypotheses with our study: (1) racial and ethnic minority populations would experience higher age-adjusted COVID-19 mortality rates than the non-Hispanic White population; (2) within racial and ethnic groups, age-adjusted COVID-19 mortality rates would be highest among those with the lowest educational attainment; and (3) racial and ethnic inequalities in COVID-19 mortality rates would remain when comparing within levels of educational attainment.

## Methods

We conducted cross-sectional analyses of cumulative COVID-19 mortality rates for US population aged 25 years or older. Although data on younger individuals were available, we excluded them for 2 reasons. First, there were a small number of deaths among those aged 24 years or younger (n = 714; <0.3% of all deaths). Second, educational attainment may not be a valid indicator of socioeconomic position for this group, as many are too young to have been able to complete schooling. Reflecting this concern, the US Census Bureau’s own analyses of education data typically exclude those younger than 25 years of age.^6^ This study analyzed solely deidentified, public-use data and was therefore exempt from institutional review board approval. This study followed the Strengthening the Reporting of Observational Studies in Epidemiology (STROBE) reporting guidelines for cross-sectional studies.^7^

We quantified racial and ethnic inequity as MRRs, both overall and within educational attainment categories. The analyses were conducted separately for each age-sex subgroup. Finally, we calculated the number of COVID-19 deaths that would have occurred if everyone had experienced the same mortality rate as college-educated non-Hispanic White individuals (the group that theoretically has the most racialized socioeconomic privilege) of the same age and sex. All analyses were based on publicly available US Census and US mortality data.

### Data Sources

We analyzed open access COVID-19 mortality data provided by the US Centers for Disease Control and Prevention (CDC).^8^ The data set is based on reporting by the 50 states and the District of Columbia for the full calendar year 2020 and reported to CDC by February 24, 2021. This data set represents the most recent available data. The file includes counts of deaths due to COVID-19 stratified by race and ethnicity, age group, sex, and educational attainment (reported as 3 categories: high school or General Educational Development (GED) certification or less; some college, which includes an associate’s degree; or a bachelor’s degree or more). We treated educational attainment as a measure of socioeconomic position; other measures such as income, wealth, and occupation were not provided in the data set and are not as readily available on death certificate–based mortality reporting systems.^9^ The CDC notes that the data set is provisional because there may be undercounts in later weeks due to reporting lags—however, the data were current as of 8 weeks past December 31, 2020, so the effect of lags should be minimal.

Sociodemographic data are reported on death certificates, typically by the funeral director. Sex was reported as “male” and “female,” with no data on whether decedents were nonbinary or transgender. Race and ethnicity were reported as “Hispanic” (Latinx; in combination with any race), as well as non-Hispanic racial groups: non-Hispanic Black, non-Hispanic White, non-Hispanic Asian, non-Hispanic American Indian or Alaska Native, and non-Hispanic Native Hawaiian and Other Pacific Islander. We treated the small proportion of decedents (<0.3%) whose race and ethnicity was categorized as “non-Hispanic more than one race” as missing and excluded them from subsequent analyses. We additionally obtained census microdata from the US Census American Community Survey for the period from 2017 to 2019.^10^ We tabulated these data for use as population denominators for all cumulative mortality rate calculations.

### Statistical Analysis

We calculated cumulative mortality rates for COVID-19 by race and ethnicity, sex, and educational attainment for the population aged 25 years or older. We conducted analyses for the entire population aged 25 years or older, as well as for the younger (25-64 years) and older (≥65 years) populations. When calculating rates for the overall, younger, and older populations, we applied direct standardization using the *dstdize* command in Stata, version 16 (StataCorp LLC) based on the CDC year 2000 standard population.^11^ To compare racial and ethnic inequality within educational attainment categories, we calculated age-adjusted cumulative MRRs comparing COVID-19 death rates for each racial and ethnic group with non-Hispanic White individuals of the same age group, sex, and education group.

In addition, we simulated a counterfactual scenario to estimate the number of deaths that would have occurred had each population group experienced the same cumulative mortality rate as the group that, theoretically, has the most racialized socioeconomic privilege: college-educated non-Hispanic White individuals. We did this by multiplying each stratum’s populations size by the mortality rate observed among college-educated non-Hispanic White individuals of the same age and sex.

All 95% CIs for cumulative mortality rates and MRRs reported below assume that mortality rates follow a Poisson distribution and are calculated using standard formulas for directly standardized rates and rate ratios.^12,13^ However, the 95% CIs represent uncertainty owing to sampling, but the mortality data represent a finite population (ie, all deaths attributed to COVID-19 in the United States), which are not subject to sampling error. Confidence intervals for MRRs that include the null value of 1.0 should therefore not be interpreted as “no statistically significant difference.” We include uncertainty estimates by convention, but focus our interpretations on the point estimates of rates and MRRs.

## Results

**Figure 1.**
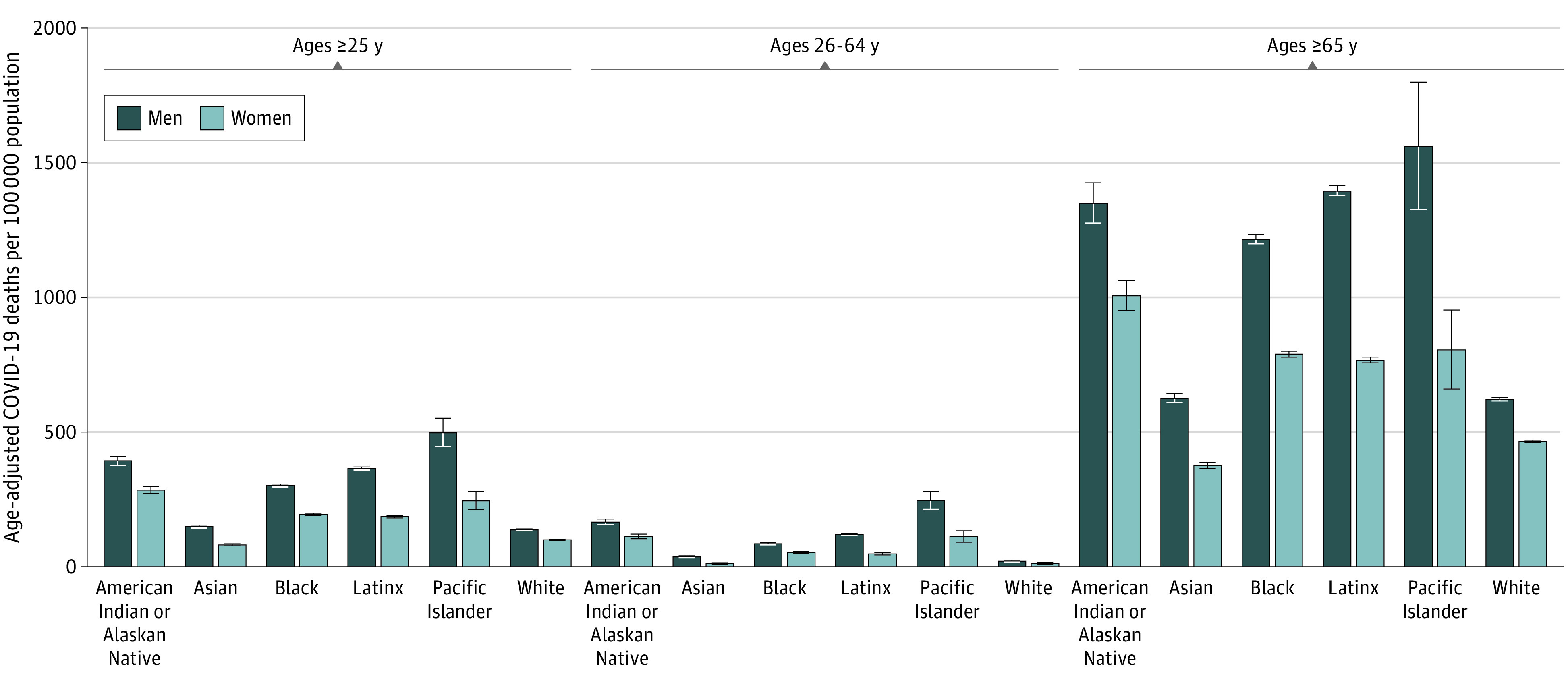
Cumulative Mortality Rates for COVID-19 in the US by Race and Ethnicity and Sex (2020) Error bars indicate 95% CIs.

Among 219.1 million adults aged 25 years or older (113.3 million women [51.7%]; mean [SD] age, 51.3 [16.8] years), 376 125 individuals ages 25 years or older died of COVID-19 during the year 2020 (Table). Among these decedents, missingness was less than 2% for educational attainment and less than 1% for race and ethnicity. Age-adjusted cumulative mortality rates for the overall population were highest among persons with the lowest educational attainment (208.1 per 100 000 population [95% CI, 207.3-208.9 per 100 000 population]). Within racial and ethnic groups, mortality rates were highest among American Indian or Alaska Native individuals (334.5 per 100 000 population [95% CI, 324.2-344.7 per 100 000 population]) and Native Hawaiian and Other Pacific Islander individuals (356.9 per 100 000 population [95% CI, 327.6-386.2 per 100 000 population]) and lowest among non-Hispanic White individuals (116.4 per 100 000 population [95% CI, 115.9-116.8 per 100 000 population]) and non-Hispanic Asian individuals (110.9 per 100 000 population [95% CI, 108.9-112.8 per 100 000 population]) (Figure 1). Racial and ethnic minority women of died at higher rates than non-Hispanic White men of the same age group, with the exception of non-Hispanic Asian women.

**Table.** COVID-19 Cumulative Deaths and Mortality Rates by Select Characteristics (2020)

Characteristic	Deaths, No.	Population, millions	Cumulative mortality rate per 100 000 population (95% CI)	
			Crude	Adjusted for age^a^
Total	376 125	219.1	116.2 (115.7-116.7)	145.9 (145.5-146.4)
Age group, y^b^				
25-39	5023	65.0	7.7 (7.5-7.9)	NA
40-54	21 896	60.5	36.2 (35.7-36.7)	NA
55-64	44 565	41.7	106.9 (105.9. 107.9)	NA
65-74	80 413	30.3	265.4 (263.6-267.2)	NA
≥75	224 228	21.6	1038.1 (1033.8-1042.4)	NA
Missing	0	NA	NA	NA
Sex				
Women	172 124	113.3	151.9 (151.2-152.6)	119.3 (118.7-119.8)
Men	204 715	105.9	193.3 (192.5-194.1)	178.6 (177.8-179.4)
Missing	0	NA	NA	NA
Race and ethnicity^c^				
American Indian or Alaska Native	4474	1.4	322.0 (312.6-331.4)	334.5 (324.2-344.7)
Asian	13 346	13.1	101.8 (100.1-103.5)	110.9 (108.9-112.8)
Black	59 528	26.2	226.9 (225.0-228.7)	237.9 (235.9-239.9)
Hawaiian and Other Pacific Islander	679	0.2	297.1 (274.8-319.5)	356.9 (327.6-386.2)
Latinx or Hispanic	68 577	34.2	200.3 (198.8-201.8)	265.2 (263.1-267.2)
White	227 532	143.9	158.1 (157.4-158.7)	116.4 (115.9-116.8)
Missing^d^	2703	NA	NA	NA
Educational attainment				
≤High school or GED	247 745	85.0	289.8 (290.3-292.6)	208.1 (207.3-208.9)
Some college	61 116	62.9	96.5 (96.4-97.9)	97.1 (96.3-97.9)
≥Bachelor’s degree	57 711	71.3	80.5 (80.3-81.6)	89.3 (88.6-90.0)
Missing	10 267	NA	NA	NA

Abbreviations: GED, General Educational Development certification; NA, not applicable.

^a^ Age-adjusted rates based on the 2000 standard US population, and numerators include complete cases only.

^b^ Age group–specific rates are not further adjusted for age.

^c^ Groups other than Latinx or Hispanic are non-Hispanic.

^d^ Includes 1124 decedents identified as having more than 1 non-Hispanic race and ethnicity.

**Figure 2.**
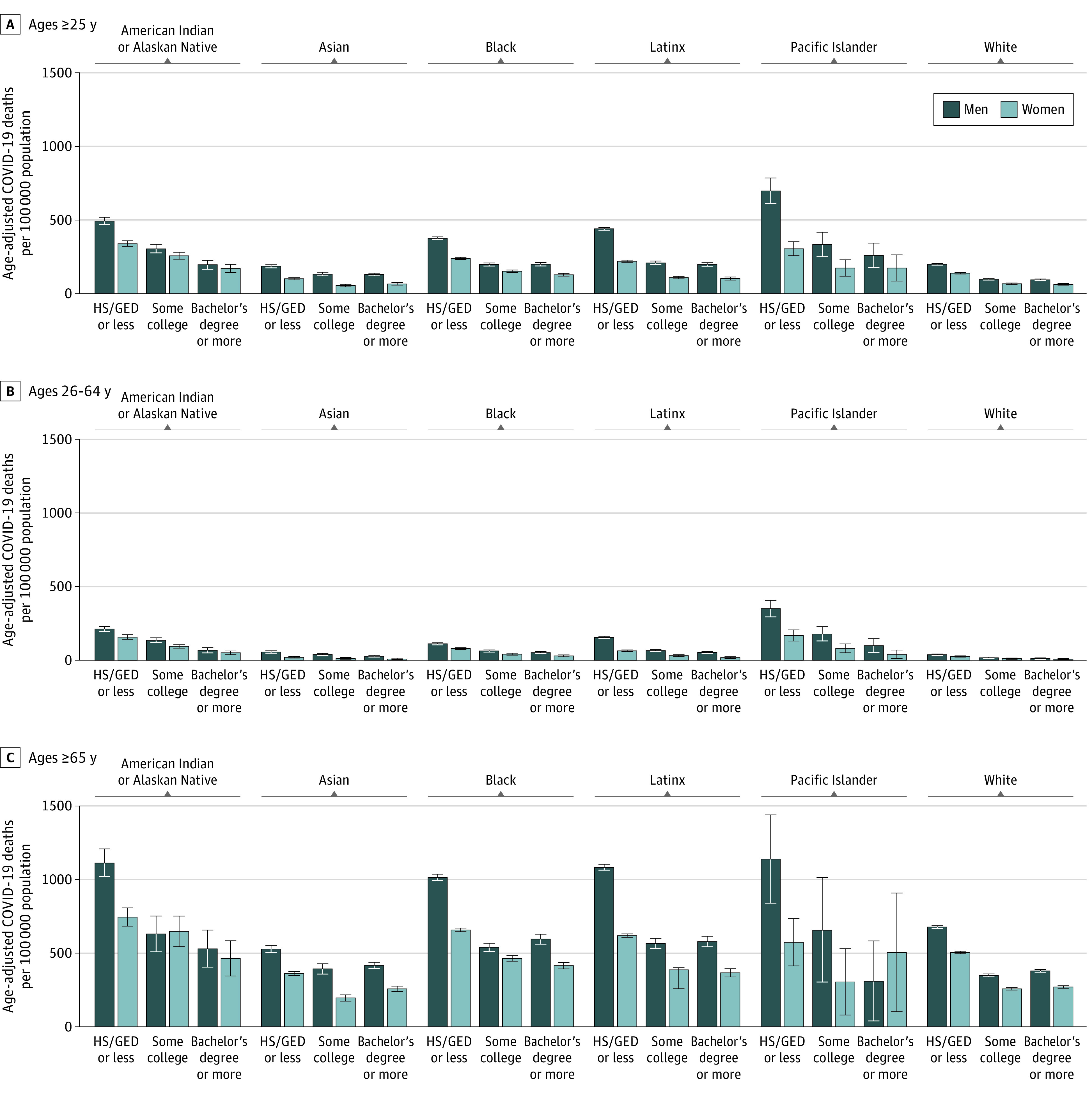
Cumulative Mortality Rates for COVID-19 in the US by Race and Ethnicity, Sex, and Educational Attainment (2020) HS/GED indicates high school or General Educational Development certification. Error bars indicate 95% CIs.

Age-adjusted cumulative mortality rates per 100 000 population ranged from 54.4 (95% CI, 49.8-59.0 per 100 000 population) among Asian women with some college to 699.0 (95% CI, 612.9-785.0 per 100 000 population) among Native Hawaiian and Other Pacific Islander men with a high school degree or less. Within race-gender groups, the highest age-adjusted cumulative mortality rates were consistently experienced by those with the lowest educational attainment (Figure 2). For the population aged 25 years or older, non-Hispanic White men with the least education died at a rate of 199.7 per 100 000 population (95% CI, 198.2-201.3 per 100 000 population), similar to the rates of college-educated non-Hispanic Black men (199.4 per 100 000 population [95% CI, 192.0-206.8 per 100 000 population]), college-educated American Indian or Alaska Native men (196.3 per 100 000 population [95% CI, 166.3-226.3 per 100 000 population]), and college-educated Latino men (198.6 per 100 000 population [95% CI, 190.7-206.5 per 100 000 population]) and lower than that of college-educated Native Hawaiian and Other Pacific Islander men (259.6 per 100 000 population [95% CI, 175.5-343.6 per 100 000 population]).

**Figure 3.**
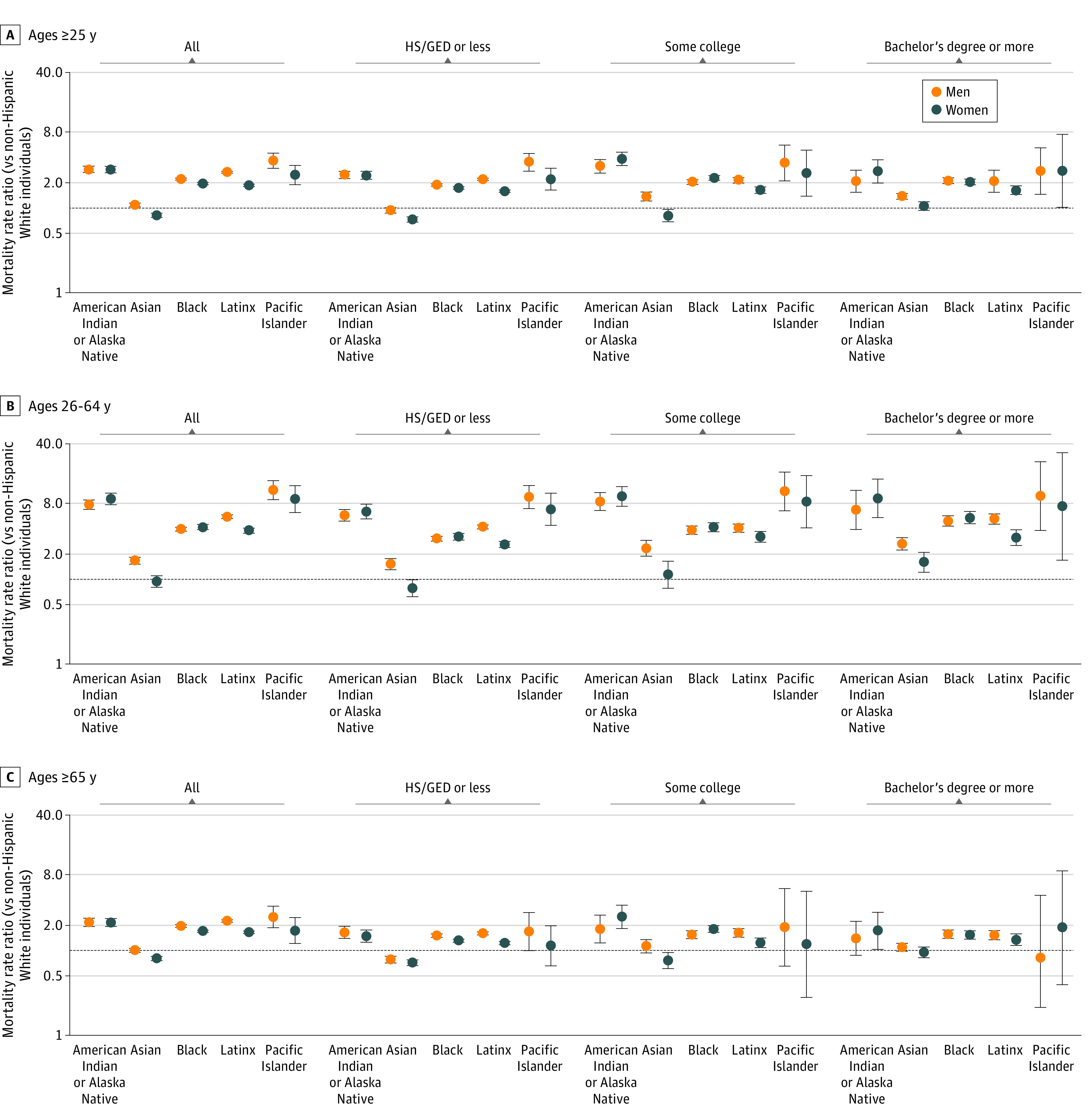
Cumulative Mortality Rate Ratios for COVID-19 in the US by Race and Ethnicity, Sex, and Education (2020) HS/GED indicates high school or General Educational Development certification. The black horizontal line at 1.0 indicates the mortality rate among non-Hispanic White individuals. Error bars indicate 95% CIs.

Nearly all racial and ethnic minority subgroups (54 of 60 age-sex-race-education subgroups, with age strata defined as 25-64 years or ≥65 years) experienced higher mortality (MRR >1.0) than their non-Hispanic White counterparts (Figure 3). The only groups with lower mortality than non-Hispanic White individuals were: older non-Hispanic Asian women of all 3 education levels, younger non-Hispanic Asian women in the lowest education category, older non-Hispanic Asian men in the highest education category, and older Native Hawaiian and Other Pacific Islander men in the highest education category. Although death was relatively rare among younger adults, the MRRs measuring racial and ethnic inequity were highest among this age group, ranging from 0.8 (95% CI, 0.6-0.1) for non-Hispanic Asian women with the least education to 11.1 (95% CI, 6.5-18.9) for Native Hawaiian and Other Pacific Islander men with some college.

Racial and ethnic inequality in mortality for the overall population remained and was slightly attenuated, on average, when comparing within education categories. For the results presented in Figure 3, the median within-education MRR was 17% lower (IQR, 0%-25%) than the MRR for all education categories combined. In some cases, the within-education MRR was larger than the overall MRR. For example, younger non-Hispanic Black women died at 4.2 (95% CI, 3.9-4.5) times the rate of younger non-Hispanic White women. Within education categories, the MRR for younger non-Hispanic Black women (vs younger non-Hispanic White women of the same education level), ranged from 3.2 (95% CI, 2.9-3.5) for those with the lowest education level to 5.4 (95% CI, 4.6-6.4) for those with the highest education level.

In a counterfactual scenario in which all people experienced the same mortality rates as college-educated non-Hispanic White individuals of the same age and sex, the total number of deaths due to COVID-19 would have been 48% lower among adults aged 25 years or older, preventing 176 000 of the 364 000 deaths for which complete sociodemographic data were available. For all racial and ethnic minority individuals, the number of deaths due to COVID-19 would have been 71% lower, preventing 100 000 deaths out of 141 000. For racial and ethnic minority individuals aged 25 to 64 years, the number of deaths would have been 89% lower, preventing 40 000 deaths out of 45 000.

## Discussion

Public-access data covering the entire United States have recently become available to examine inequities in COVID-19 mortality jointly by race and ethnicity and socioeconomic position. Our study of population-based data among adults aged 25 years or older identified inequities by educational attainment for the overall population and within racial and ethnic groups. Racial and ethnic minority populations typically experienced higher age-adjusted mortality rates than non-Hispanic White individuals and those with the lowest socioeconomic position (high school or GED completion or less) also died at the highest rates within racial and ethnic groups. The socioeconomic privilege afforded by higher educational attainment among racial and ethnic minority populations was often insufficient to overcome racial and ethnic inequality. For example, adjusting for age, college-educated non-Hispanic Black men had higher COVID-19 mortality rates than non-Hispanic White men who had completed high school or GED or less. Although a gradient in mortality rates by educational attainment was found within all population groups, the association of unequal educational attainment with racial and ethnic inequalities in COVID-19 deaths was likely modest.

Racialized socioeconomic inequities played a substantial role in the overall death toll of the pandemic. Had all population groups experienced the mortality rates observed among the group assumed to be the most racially and socioeconomically privileged (college-educated non-Hispanic White individuals), the overall number of COVID-19 deaths in the US during 2020 would have been halved, and deaths among racial and ethnic minorities aged 25 to 64 years would have been reduced to about one-tenth of the observed number.

One of our key findings—that the magnitude of racial inequities was only slightly attenuated on average when stratifying by educational attainment categories—warrants further explanation and, ultimately, more exploration in future research. Racial and ethnic differences in educational attainment, and in socioeconomic position more broadly, capture only one mechanism through which structural racism is associated with health. Prior research shows that racial and ethnic differences in economic assets vary considerably even within education levels. For example, non-Hispanic Black college graduates in the US have less wealth and lower rates of home ownership when compared with non-Hispanic White college graduates and even when compared with non-Hispanic White indiviuals who have not graduated high school.^14,15^ With regard to COVID-19 specifically, census data show all racial and ethnic groups other than non-Hispanic White individuals are more likely to have risk factors for SARS-CoV-2 exposure (household crowding, multigenerational housing, and potential occupational and paraoccupational exposure owing to employment in high-risk jobs or cohabitation with such workers) than non-Hispanic White individuals of the same educational attainment category (eFigure in the Supplement). Prior research suggests that racial and ethnic minority populations have been infected with COVID-19 at far higher rates than non-Hispanic White individuals,^16,17,18^ and that inequities in COVID-19 mortality rates may in large part be associated with these differences in exposure to the virus.^19^ Although there has been a small number of studies exploring the degree to which racial and ethnic differences in COVID-19 outcomes may be associated with differences in population genetics, the evidence for this has so far been very limited.^20^ In addition, racial and ethnic groups that do not share geographic ancestry (eg, American Indian and Pacific Islander) have nevertheless experienced the highest COVID-19 mortality rates, suggesting that similarities in COVID-19 outcomes are associated more with similar social conditions than population genetics.

Different measures of socioeconomic position such as wealth or income may have yielded different patterns of racial inequality. In addition, accounting for social class, which, unlike socioeconomic position, is defined by one’s position in economic relationships (eg, as a worker or owner),^9^ may be particularly useful in analyses of COVID-19 outcomes, because class is closely tied to power—in this case, power to mitigate exposure to SARS-CoV-2.

### Limitations

This study has some limitations. The analyses in this study relied on race and ethnicity classification of decedents from death certificates, which prior research has demonstrated to substantially underestimate mortality for American Indian or Alaska Native populations.^21^ It is unclear whether similar underestimation of mortality also applies to Native Hawaiian and Other Pacific Islander individuals, as they have not been disaggregated from non-Hispanic Asian individuals in prior research on misclassification of race and ethnicity in mortality data. In addition, COVID-19 is likely misclassified in mortality data, and the CDC estimates that the true number of COVID-19 deaths was 30% higher than reported for the period from March 2020 to May 2021,^22^ although whether misclassification varies by sociodemographic groups is unknown. As this was a cross-sectional study, we were also unable to assess whether the magnitude of inequalities changed over time. Finally, owing to limitations in the mortality data set, we were not able to assess potential mediators associated with racial and ethnic and socioeconomic inequalities, including geography, SARS-CoV-2 exposure, health care access, and comorbidities.

## Conclusions

During the first year of the COVID-19 pandemic, which largely preceded the availability of vaccines, there were extremely high levels of racialized economic inequity in the distribution of COVID-19 mortality. Future research may investigate the specific pathways that produced these joint racial and ethnic and socioeconomic inequities, as well as whether the longstanding political disempowerment of populations among whom the virus was most lethal (ie, economically marginalized racial and ethnic minority groups) was associated with policy responses to the pandemic. What is clear is that the mortality burden of these inequities is high. Future research can help inform interventions that yield equitable responses to both the ongoing burden of COVID-19 and potential future pandemics that spread similarly to SARS-CoV-2.

## Notes

Supplement. **eFigure A.** Distribution of Household Crowding (>1 Person/Room) **eFigure B.** Distribution of Employment in Exposure Occupations and/or Household Exposure to Such Workers **eFigure C.** Multigenerational Housing Among Those Ages ≥65 Years
