# Supplementary material for: Variation in COVID-19 Mortality in the US by Race and Ethnicity and Educational Attainment
Source: JAMA Netw Open. 2021 Nov 23;4(11):e2135967. doi: 10.1001/jamanetworkopen.2021.35967 (PMC8611482; doi:10.1001/jamanetworkopen.2021.35967)
Supplement: Supplement. — eFigure A. Distribution of Household Crowding (>1 Person/Room) eFigure B. Distribution of Employment in Exposure Occupations and/or Household Exposure to Such Workers eFigure C. Multigenerational Housing Among Those Ages ≥65 Years [file jamanetwopen-e2135967-s001.pdf]

Supplementary Online Content

Feldman JM, Bassett MT. Variation in COVID-19 mortality in the US by race and ethnicity and educational attainment. *JAMA Netw Open.* 2021;4(11):e2135967. doi:10.1001/jamanetworkopen.2021.35967

eFigure A. Distribution of Household Crowding (>1 Person/Room)
eFigure B. Distribution of Employment in Exposure Occupations and/or Household Exposure to Such Workers
eFigure C. Multigenerational Housing Among Those Ages ≥65 Years

This supplementary material has been provided by the authors to give readers additional information about their work.

© 2021 Feldman JM et al. *JAMA Network Open.*

Data are from the authors' analysis of US Census American Community Survey microdata (2017–2019. Characteristics are based on self-reported data. For Supplementary Figure B, households with high SARS-CoV-2 exposure occupation members were identified based on the 20 highest-mortality occupations reported by Chen Y-H, Glymour M, Riley A, et al. Excess mortality associated with the COVID-19 pandemic among Californians 18–65 years of age, by occupational sector and occupation: March through October 2020. *medRxiv*. Published online January 22, 2021:2021.01.21.21250266. doi:10.1101/2021.01.21.21250266
.

eFigure A. Distribution of Household Crowding (>1 Person/Room)

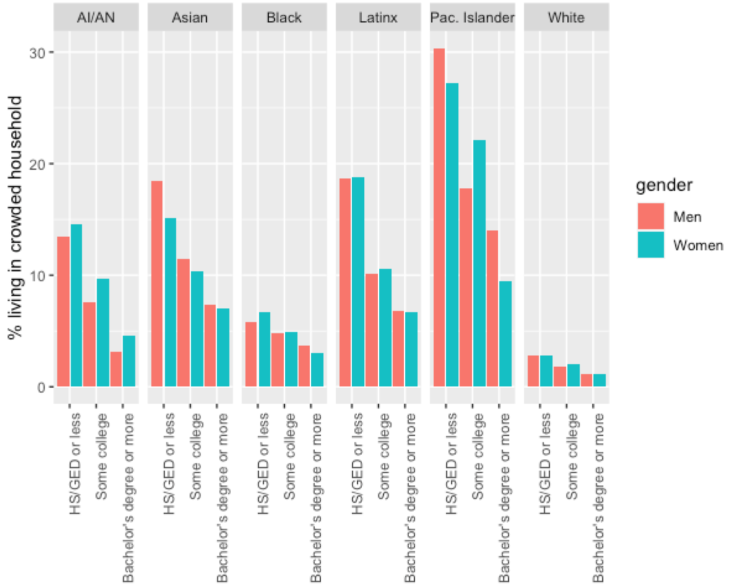

eFigure B. Distribution of Employment in Exposure Occupations and/or Household Exposure to Such Workers

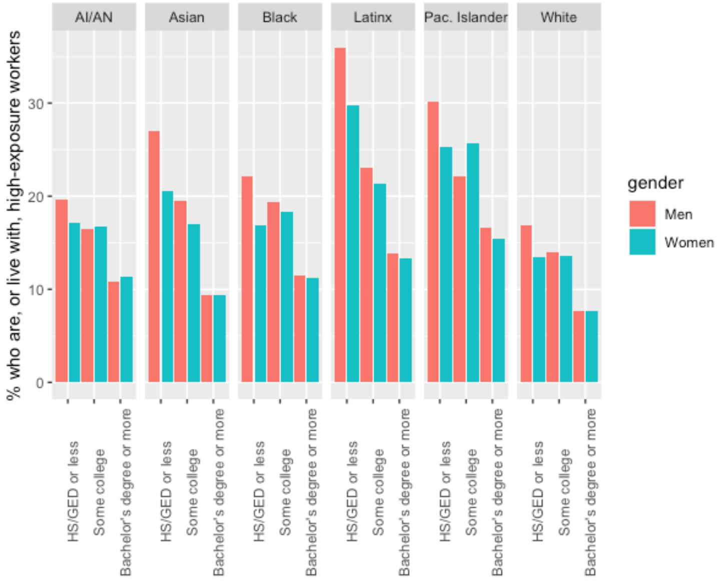

© 2021 Feldman JM et al. *JAMA Network Open.*

eFigure C. Multigenerational Housing Among Those Ages ≥65 Years

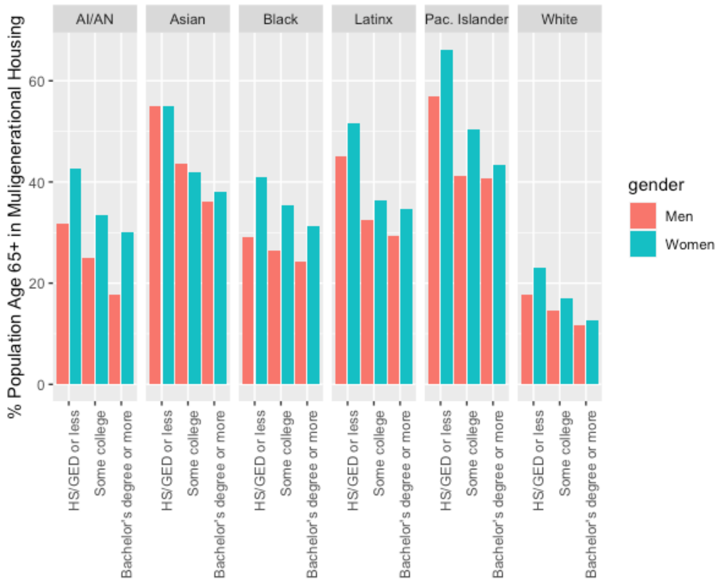

© 2021 Feldman JM et al. *JAMA Network Open.*
